# Quality indicators and patient outcome measures for palliative care in cancer patients: a systematic review

**DOI:** 10.3332/ecancer.2025.1929

**Published:** 2025-06-20

**Authors:** Chase Peng Yun Ng, Moira Hegyi, Grant Lewison, Tania Pastrana, Eve Namisango, James Cleary, Barbara Hasties, Eric Kabisa, Helena Musau, Kathryn Spangenberg, Paola Ruiz, Zipporah Ali, Mertixell Mallafre-Larrosa, Alfredo Polo, Julie Torode, Ajay Aggarwal, Richard Sullivan, Mevhibe Hocaoglu

**Affiliations:** 1 King’s College London, Institute of Cancer Policy, London WC2R 2LS, UK; 2 Department of Palliative Medicine, Medical Faculty RWTH Aachen University, Aachen 52062, Germany; 3 African Palliative Care Association, Makindye, Kampala, Uganda; 4 Supportive Oncology, Department of Medicine, Division of Hematology/Oncology, Indiana University School of Medicine, Indianapolis, IN 46202, USA; 5 Team Humanity International, Amsterdam 1082MT, The Netherlands; 6 Rwanda Palliative Care and Hospice Organization (RPCHO), Kigali, Rwanda; 7 Kenyatta University Teaching Research and Referral Hospital, Box 7674 - 00100, GPO, Nairobi, Kenya; 8 Komfo Anokye Teaching Hospital, Box 1934, Adum-Kumasi, Ghana; 9 La Asociación Cuidados Paliativos de Colombia ASOCUPAC, Cali, Colombia; 10 Kenya Hospices and Palliative Care association (KEHPCA), Nairobi, 20854-00202 Kenya; 11 City Cancer Challenge, Geneva, 1204 Switzerland; 12 Cicely Saunders Institute King’s College London, London SE5 9RS, UK; 13 Health Services Research & Policy, London School of Hygiene & Tropical Medicine, London WC1E 7HT, UK

**Keywords:** palliative care, quality indicator, outcome measure, cancer, global oncology, structure of care, process of care

## Abstract

**Introduction:**

With the exponential rise in global cancer incidence, the surge in demand for palliative care has outstripped capacity, limiting patients’ access to quality and holistic palliative care, especially in low- and middle-income countries. Despite an upturn in research activity, evidence in palliative care remains limited, given its complexity as well as the shortage of standardised quality indicators (QIs) and patient outcome measures (POMs). The objective of this systematic review is to assess the QIs and POMs used to evaluate palliative care service on aggregated and individual levels.

**Methods:**

We undertook a systematic review following the Preferred Reporting Items for Systematic Reviews and Meta-analysis guidelines to determine the QIs and/or POMs of palliative care in patients with non-communicable diseases. A Web of Science, EMBASE, PubMed and SOCSCI search between 1 January 2013 and 31 Dec 2022 identified 41 articles. We appraised the quality of all studies using the mixed methods appraisal tool.

**Results:**

26.8% of studies focus on QIs, while 73.2% used POMs. >90% of palliative care research took place in high-income settings. Across domains of palliative care, the outcome of care is most studied, while the structure and process of palliative care are understudied. QIs and POMs identified often had overlapping themes. Due to the multidimensionality and intricacy of palliative care, evidence is limited, patchy and heterogenous in quality.

**Discussion:**

There is an overall lack of standardisation of QIs and POMs, as well as variability in evidence of palliative care research. We recommend that stakeholders collaborate to develop a standardised repository of metrics for monitoring and evaluating palliative care services at both individual and system levels, with a particular focus on structural and process indicators. Incorporating validated, patient-centred measures and selecting key items as quality indicators will enable meaningful tracking of changes, guiding resource allocation and driving improvements in patient-centred care. Furthermore, exploring alternative research designs is essential to enhance feasibility, uphold ethical integrity and strengthen the robustness of future studies.

## Introduction

Cancer remains a worldwide public health challenge, despite the growing investments in research and development towards preventative and treatment interventions [1]. The global cancer incidence is projected to reach 30.2 million new cases and 16.3 million cancer-related deaths by the year 2040 [2, 3] Concurrently, the need for palliative care over the years has increased and it has been estimated that globally 40 million people currently require palliative care [4]. However, only about 14% of this number receive palliative care services [5].

Palliative care is defined as care that improves the quality of life of patients and that of their families who are facing challenges associated with life-threatening illness, whether physical, psychological, social or spiritual [5] Over the years, international health groups, such as the World Health Organisation (WHO), non-governmental organisations and governments have increasingly prioritised access to palliative care for cancer patients, especially in low- and middle-income countries (LMICs) [6, 7]. This attention has stimulated research activities in palliative medicine. According to a systematic review conducted in 2008, the proportion of palliative care and hospice research publications from all Ovid Medline publications rose from 0.08% in 1970 to 0.38% in 2005 [8] A recent publication looking at global palliative care research using a bibliometric review and mapping analysis quantified that palliative care publications increased by around fourfold from 2002 to 2020, with a 19% 5-year increase projected in 2025 [9].

Despite the rise in research output, evidence on measuring the scope of need and effectiveness of palliative care remains limited and of low quality [8, 10]. The paucity of evidence could be attributed to the lack of standardised quality indicators (QIs) and patient outcome measure (POM) in palliative care. QIs are defined, measurable items referring to the outcome, process or structure of care for a particular type of patient or clinical circumstance on an aggregated level [11]. They are often described with a numerator, a denominator and a performance standard, which form the benchmark for healthcare systems to evaluate and monitor their palliative care service holistically. On the other hand, POMs are focused on patient- or family-level status and response to symptoms and conditions in all domains on an individual level [12].

To address this problem, the National Coalition for Hospice and Palliative Care published the fourth edition of the National Consensus Project Clinical Practice (NCP) Guidelines for Quality Palliative Care in 2018 with the aim to standardise and outline evidence-based processes and practices of safe and reliable high-quality palliative care in all settings [13]. To ensure that palliative care is inclusive, the guideline divides palliative care into eight domains: 1. structure and process of care; 2. physical aspects of care; 3. psychological and psychiatric aspects of care; 4. social aspects of care; 5. spiritual, religious and existential aspects of care; 6. cultural aspects of care; 7. care of the patient nearing the end of life and 8. ethical and legal aspects of care [14]. While the effort is commendable and progressive, a systematic review led by Ahluwalia *et al* [15] revealed that the quality of evidence supporting the guideline remains low despite substantial support in clinical practice [15]. Another systematic review of palliative care assessment tools using the eight domains identified, led by Aslakson *et al* [16], further highlighted the nonuniform application and the shortage of QIs in evaluating the structure and process of palliative care.

In the field of oncology, the European Society of Medical Oncology (ESMO) had recognised this problem since 2003 and promoted formal integration of oncology and palliative care services based on WHO recommendations through an accreditation programme, focusing on credentialling, education, training and research of palliative care in addition to service provision[17]. It created QIs for hospitals to compare the structure and process of their palliative care service on a systemic level, thus complementing the NCP guidelines which focus on the different aspects of patient outcome. In this review, we adapted the domains from both institutions to ensure that all structure, process and outcome of care are adequately represented and evaluated to inform future palliative care practice holistically.

As palliative care is often translatable in practice across cancer patients and patients with other non-communicable diseases, the objectives of our systematic review were to evaluate the palliative care QIs and POMs for patients living with all non-communicable diseases. We aimed to identify gaps in quality assessment of the palliative care service, especially in cancer care delivery across healthcare systems in different income settings, and to highlight validated QIs and POMs that have the potential to be used as standards in wider settings, especially for cancer patients in the LMICs.

## Methods

Our approach was guided by the Arksey and O’Malley [18] framework and supported by the Levac *et al*. [19] recommendation. The Arksey and O’Malley [18] framework comprised the following: (i) identifying the research question, (ii) identifying relevant studies, (iii) study selection, (iv) charting the data and (v) collating, summarising and reporting the results and (vi) consultation. An optional sixth stage of consultation was proposed by Arksey and O’Malley [18] as a measure to seek insight from stakeholders beyond what was found in the literature [17]. This scoping review included consultation with an expert reference group of stakeholders as we were interested in both the palliative care QIs and results/functionality of POMs [19].

### Identification of relevant studies

The systematic review was designed using the Preferred Reporting Items for Systematic Reviews and Meta-analysis (PRISMA). Studies published between 1st January 2013 and 31st December 2022 were searched on 4 online databases using Web of Science, EMBASE, PUBMED and SOCSCI using a complex bibliometric filter (Appendix A). The search terms were agreed upon following two consensus meetings of the expert reference group to identify relevant publications reflecting palliative care and quality indicators/ measures for both cancer and non-cancer patients globally.

### Inclusion criteria

The inclusion criteria were as follows: any qualitative, quantitative and mixed-method studies on QIs and POMs of palliative care relevant to all types of chronic non-communicable diseases in English language.

### Exclusion criteria

Studies with the sole therapeutic intent of prolonging life expectancy and not the quality of life of patients were excluded. Studies that did not use any qualitative or quantitative quality or patient-level outcome measures of palliative care were also excluded. Review articles, case reports, letters, abstracts, conference proceedings, editorials, expert opinions, preclinical studies, protocols and laboratories studies were excluded.

### Data selection

The titles and abstracts were assessed by two independent reviewers, Peng Yun Ng (PN) and Moira Hegyi (MH), to minimise reviewer errors and bias. All identified abstracts underwent full-text review by PN and MH. PN and MH extracted data from each included study, assessing its quality and any uncertainties or discrepancies were resolved through discussion with RS and AA.

### Data extraction

The data included in the extraction are as follows:

- Location of study
- Characteristics of study (funding, research design, sample size, aim and outcome of study)
- Disease (cancer or non-cancer chronic non-communicable disease)
- Type of palliative care service (home care, inpatient care, outpatient care, hospice, bereavement support and caregiver support)
- Purpose of palliative care intervention (education, research and service provision)
- Domain of palliative care service
  - Structure of care: human resource, facilities and equipment organisational
  - Outcome of care: physical, psychological, social, spiritual, ethics and legal
  - Process of care
  - Type of quality indicators/patient outcome measures

### Quality appraisal of included studies

To assess the quality of the included studies, we used the mixed methods appraisal tool (MMAT), version 2018 [20]. The quality appraisal procedure was carried out by two independent reviewers, PN and MH. The quality of the evidence will be graded using the following percentage scores: (1) 0%–50% will represent low-quality evidence, (2) 51%–75% will represent average quality evidence and (3) 76%–100% will represent good quality evidence. We evaluated various study methodologies including qualitative, quantitative and mixed-methods studies, using this quality appraisal method. We summarised the detailed breakdown of the quality appraisal of each study in a table.

## Results

### Search strategy

**Figure 1.**
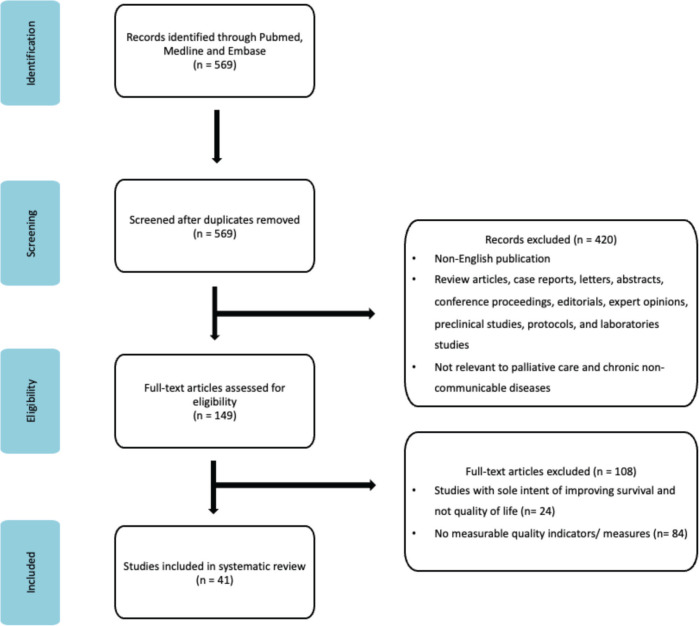
PRISMA flow chart of identification for articles for inclusion.

569 publications potentially relevant to QIs and POMs for palliative care were initially identified by the complex bibliometric filter. After the abstracts and titles were screened, 420 studies were not included due to them being (1) review articles, case reports, letters, abstract, conference proceeding, editorials, expert opinions, preclinical studies, protocols and laboratories studies, (2) not relevant to palliative care and chronic non-communicable disease and (3) non-English. Of the remaining 149 studies, a further 107 studies were excluded as 24 studies focused solely on improving the survival of the patients instead of their quality of life; and 84 studies did not utilise any measurable palliative care QIs or POMs. 41 publications fulfilled the search criteria and are summarised in Tables S1 and S2 (Appendix A and B). The search strategy is illustrated in Figure 1.

## Palliative care research activity

### Disease

58.5% (*n* = 24) of studies focused on cancer patient [21, 22, 24, 25, 27–29, 31, 32, 34, 35, 37, 38, 40–45, 49, 54, 56, 57, 59], while 41.5% (*n* = 17) focused on non-cancer NCD such as dementia, chronic kidney disease and Parkinson’s [26, 30, 33, 36, 39, 46–48, 50–53, 55, 58, 60, 61]. There were 26.8% (*n* = 11) true quality indicator studies [21–31] and 73.2% (*n* = 30) patient outcome measures [32–61].

### Methodology

85.4% of the studies (*n* = 35) were quantitative [21–24, 26–34, 36, 38–40, 42–46, 48–51, 53–61]; 11.9% (*n* = 5), qualitative [25, 35, 37, 41, 47] and 2.4% (*n* = 1), mixed methods [52]. The majority of the quantitative studies were non-randomised (71.4%, *n* = 25) [21–24, 27, 28, 30, 32, 33, 36, 38–40, 42, 44–46, 49–51, 53, 56–58, 61] and descriptive (22.2%, *n* = 8), [26, 29, 31, 34, 43, 48, 55, 59] while only two of them were randomised (5.6%, *n* = 2) [54, 60].

### Location

**Figure 2.**
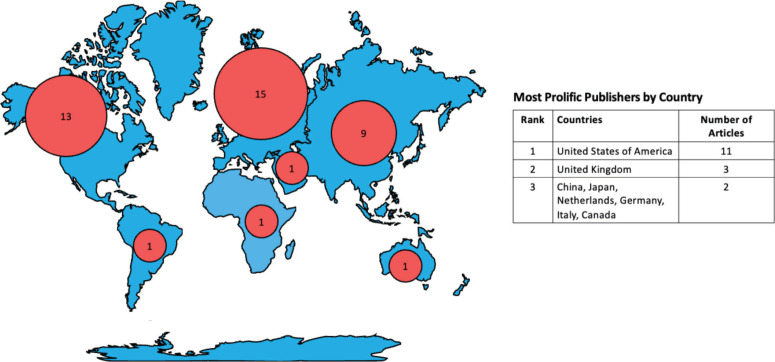
Hotspots of palliative care studies related to quality indicators and patient outcome measures by Continent and Country.

As illustrated in Figure 2, Palliative care research in quality indicators and patient outcome measures was highly concentrated in Europe, 36.6% (n = 15), [29, 34, 37, 38, 40–42, 47, 49, 51, 52, 54, 56, 57, 60] and North America, 31.7% (*n* = 13) [21, 23–28, 31, 32, 50, 58, 59, 61] especially the United States. Asia was the origin of 22.0% (n = 9) of the literature, [22, 30, 33, 36, 43–46, 55] whereas the Middle East, South America, Africa and Australia only contributed 2.4% each [35, 39, 48, 53]. Only 7.3% (n = 3) of literature was published from LMICs [35, 39, 44].

### Quality assessment

All included studies of various methodologies were evaluated and graded using the MMAT (ver 2018). The outcomes of the quality assessment are summarised in Table 1.

29 out of 41 studies (70.7%) were graded as having high-quality evidence (75%–100% score), [23, 25–27, 29–31, 33–39, 41–43, 45–52, 56, 58, 60, 61] while 12 (29.3%) studies were graded as medium quality evidence (50%–75% score) [22, 24, 25, 28, 32, 44, 49, 53–55, 57, 59]. For the 11 QI studies, the grade assessment was high for 72.7% (*n* = 8) and medium for 27.3% (*n* = 3). As none of the studies were scored as having low-quality evidence (0%–50% score), the results of the review remain unchanged with sensitivity analysis.

#### Type of palliative care services

**Figure 3.**
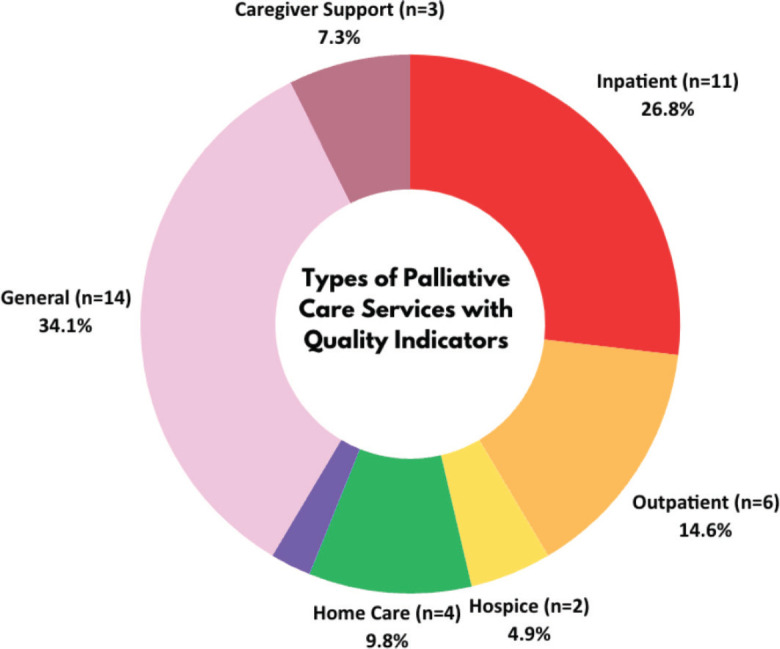
Types of palliative care services with quality indicators.

Palliative care QI and POM studies were identified across various palliative care settings, predominantly in inpatient/acute (26.8%, *n* = 11) [21, 22, 27, 29, 31, 48–50, 53, 60, 61] and outpatient care (14.6%, *n* = 6), as depicted in Figure 3 [24, 25, 32, 34, 56, 58]. They are also commonly used to evaluate the quality of palliative care in the context of home care (9.8%, *n* = 4) [26, 30, 36, 42] and caregiver support (7.3%, *n* = 3), [45, 51, 52] as well as hospice (4.9%, *n* = 2) [23, 35] and bereavement support (2.4%, *n* = 1) [47].

## Domain

**Figure 4.**
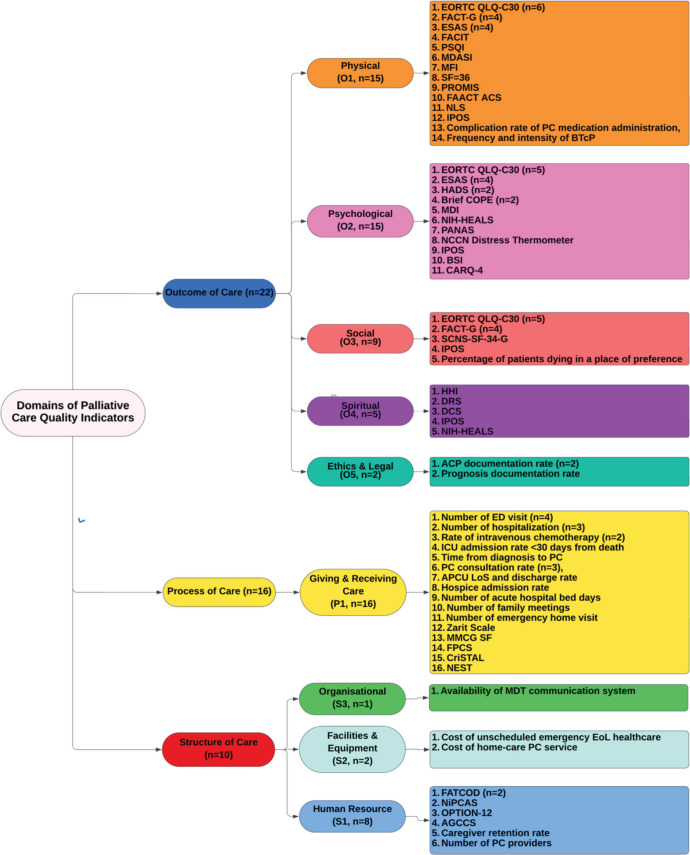
Quality indicators across domains of palliative care.

The palliative care QIs and POMs can be categorised into three domains: structure of care, process of care and outcome of care as shown in Figure 4.

FATCODS = Frommelt Attitudes Toward Care of the Dying; NipCAS = Neonatal Palliative Care Attitude Scale; OPTION-12 = Observing Patient Involvement Scale; AGCCS = Anticipatory Grief Counseling Competency Scale; PC = palliative care; MDT= Multidisciplinary Team; EoL = End of Life; APCU = Acute Palliative Care Unit; LoS = Length of Stay; MMCG = Marwit-Meuser Caregiver Grief; FPCS = Family Perceptions of Care Scale; CriSTAL = Criteria for Screening and Triaging to Appropriate aLternative care; NEST = Needs at the End-of-Life Screening Tool; ED =; FACIT = Change in Functional Assessment of Cancer Illness Therapy; FACT-G = The Functional Assessment of Cancer Therapy - General; PSQI = Pittsburg Sleep Quality Index; MDASI = M.

D. Anderson Symptom Inventory; EORTC QLQ-C30 = European Organisation for the Research and Treatment of Cancer Quality of Life Questionnaire core; MFI = Multidimensional Fatigue Inventory; SF = Short Form; PROMIS = patient-reported outcomes measurement information system; ESAS = Edmonton Symptom Assessment System; FAACT ACS = Functional Assessment of Anorexia/Cachexia Therapy Anorexia Cachexia Subscale; NLS = Nutrition Literacy Scale; IPOS = Integrated Palliative Outcome Set; BTcP = Breakthrough cancer Pain; HADS = Hospital Anxiety and Depression Scale; MDI = Major Depression Inventory; NIH-HEALS = National Institute Health Healing Experience of All Life Stressors; PANAS = Positive and Negative Affect Schedule; NCCN = National Comprehensive Cancer Network; BSI= Brief Symptom Inventory; COPE = Coping Orientation to Problems Experienced; SCNS = Supportive Care Needs Survey; HHI = Herth Hope Index; CARQ = Concerns about Cancer Recurrence Questionnaire; DRS = Decision Regret Scale; DCS = Decisional Conflict Scale; ACP = Advanced Care Planning

### Structure of care

Based on the WHO report on palliative care, structure of care could be divided into three subdomains: human resource, facilities and equipment as well as organisation. Six of the POM studies focused on human resource ranging from clinicians’ attitude to palliative care, such as from melt attitudes toward care of the dying (FATCODS) and neonatal palliative care attitude scale (NipCAS), to their competency in grief counselling and shared decision-making skills using anticipatory grief counselling competency scale (AGCCS) an observing patient involvement scale respectively [33, 39, 45, 55, 57, 58]. The remaining two papers focussed on QIs, quantifying the palliative care workforce across different services i.e. number of palliative care nurse practitioner and caregiver retention rate [25, 50]. In terms of facilities and equipment, the QIs identified were cost related to unscheduled emergency end-of-life (EoL) care, cost of home-care palliative service and number of acute hospital bed days [25, 29, 30]. It is worth noting that there were no QIs assessing the availability of palliative care medication, equipment and various services across primary, secondary and tertiary care. The only QI for organisational structure of palliative care was for the availability of a multidisciplinary communication system [25].

### Process of care

16 out of 41 studies (39.0%) used QIs and POMs to evaluate the process of PC provision [21–23, 25–28, 31, 36, 50–54, 59, 61]. The 17 QIs and POMs identified broadly fell into three categories – the rate of utilisation of PC services, caregiver burden and EoL care screening. The rate of patients approaching their end-of-life attending the emergency department or requiring emergency home visits, rate of hospitalisation, rate of admission to intensive care unit before death, rate of admission to hospice, frequency and timing of PC consult from diagnosis and rate of systemic therapy were QIs used to measure the utilisation of various types of palliative care provided. On the other hand, caregiver burden were assessed using modified caregiver strain index, Zarit Scale, Marwit-Meuser Caregiver Grief (MMCG) and family perceptions of care scale [26, 51, 52]. Except for MMCG, these POMs assessed caregivers in the form of self-administered instruments, across their financial, physical, psychological, social and personal domain. MMCG focused purely on grief and has been validated in patients with brain injury, Alzheimer’s dementia and cancer [52]. To facilitate identification of patients who might benefit from PC, Criteria for Screening and Triaging to Appropriate aLternative care (CriSTAL) and needs at the end-of-life screening tool (NEST) were utilised [53, 61]. CRiSTAL relied on patient demographics (age, comorbidities), previous admissions as well as investigations including ECG and urinalysis [53]. On the other hand, NEST was a 13-question comprehensive screening tool co-developed with patients to evaluate their needs holistically across four core themes – for Needs (social), for Existential matters, for Symptoms and for Therapeutic matters [61].

### Outcome of care

22 out of 41 studies (53.7%) assessed outcomes of palliative care [24, 26, 32, 34, 35, 37, 38, 40–44, 46–49, 51, 52, 54, 56, 59, 60]. 14 of those studies evaluated the physical symptoms and needs of the patients. The most popular POM (*n* = 6) identified was the 30-question EORTC-QLQ-C30, which focused on daily function of cancer patients and their perception of health and quality of life [34, 37, 38, 41, 42, 56]. It broadly screened patients of various physical, psychological and social care needs, but did not include spiritual and ethics-related subdomains of care. Similarly, the Functional assessment of cancer therapy (FACT-G), Edmonton Symptom Assessment System (ESAS), Change in Functional Assessment of Cancer Illness Therapy (FACIT) and M.D. Anderson Symptom Inventory (MDASI) assessed cancer patients on their physical and psychological symptoms as well as functional needs [32, 40, 59]. MDASI also contained assessments specific to different types of cancer such as Acute Myeloid Leukaemia (AML) [32]. Patient-reported Outcomes Measurement Information System (PROMIS) is a POM newly developed to standardise ways to quantify patient-reported outcomes, such as pain, fatigue, physical functioning, emotional distress and social role participation and track changes in research settings [37]. The Integrated Palliative Care Outcome Set (IPOS) covered all the same domains but further included spiritual care within its assessment [54]. On the other hand, Pittsburgh Sleep Quality Index, Multidimensional Fatigue Inventory and Functional Assessment of Anorexia/Cachexia Therapy Anorexia Cachexia Subscale focused on the specific physical symptoms of sleep, fatigue and anorexia, respectively [32, 34, 43]. Nutrition literacy scale was developed to assess patients’ knowledge of their nutrition status and requirement to optimise dietary plans [46].

The psychological subdomain was the second most commonly assessed (*n* = 12, 29.3%) with widely used and validated POMs adopted from the psychiatric field, such as Hospital Anxiety and Depression Scale and major depression inventory [32, 34, 35, 40, 41, 47, 52, 54, 56, 59, 60]. Other POMs used include the healing experience of all life stressors tool, a 35-item questionnaire developed by the NIH Clinical Centre Pain and Palliative Care Service as a psycho-social-spiritual measure of healing that quantified and tracked positive change in response to significant life events and positive and negative affect schedule, which was a psychometric scale to gauge the patients’ positive and negative affect [35, 47]. In addition, the National Comprehensive Cancer Network Distress Thermometer was developed and used for cancer patients to rate their level of distress subjectively on a weekly basis in clinical and research settings [54]. Similarly, the brief symptom inventory assessed the psychological distress of patients but it lacked validity among cancer patients, especially adolescents and young adults [56]. Coping orientation to problems experienced inventory, on the contrary, focused on the ability of patients coping emotionally and physically with the distress from the significant life events [44, 52]. Concerns about Cancer Recurrence Questionnaire-4 was used to evaluate the level of fear using the frequency, intrusiveness and degrees of distress the patients reported [34].

Social and spiritual care were the joint third most common (*n* = 6, 14.6%) subdomains [26, 32, 34, 35, 41, 42, 51, 54, 59]. For the social aspect of care, besides the aforementioned EORTC-QLQ-C30, FACT-G and IPOS, Supportive Care Needs Survey-Sort Form was used to evaluate the social care needs of cancer patients [54]. It has been validated in multiple countries and found to be culturally appropriate to inform supportive care requirements. Finally, the percentage of patients dying in their place of preference was also used as a QI to support PC [26].

Spiritually, the herth hope index was a 12-question POM tool used to quantify different unique dimensions of hope in clinical settings taking into consideration the philosophical, religious, sociological and psychological factors [32]. However, a recent systematic review revealed wide variability in its applicability and validity across cultures [62]. Decision regret scale and decisional conflict scale were designed and developed to measure the uncertainties and regret patients faced with decisions made and evaluate the quality of shared decision making [34, 51].

Ethics and legal aspects of care were the least studied (*n* = 2, 4.8%) subdomain within the outcome of care domain [24, 51]. The QIs identified in our review were the documentation rates of advanced care planning and prognosis in hospital and nursing care home settings.

## Discussion

### Lack of standardisation of quality indicators and patient outcome measures

The results of this review principally highlight the overall lack of standardisation of QIs and POMs related to palliative care. Within each of the three domains of structure, process and outcome of care, multiple QIs and POMs were used to assess the same subdomains and nearly half of the studies used multiple metrics. This can be attributed to a lack of guidance and methods to search, compare and appraise the validity and reliability of QIs and POMs designed for the same domain. POMs assessing the physical and psychological aspects of care, were by far the most prominent in the 41 studies screened. However, 15 and 11 different POMs were used within these two subdomains respectively. For example, FACT-G, ESAS and FACIT assess identical physical symptoms of patients on different subscales, deriving different outcomes from different interpretations [37, 40, 59, 60]. The lack of standardised POMs makes direct comparisons and meta-analyses difficult and hinders the possibility of using longitudinal data for intervention comparison. Furthermore, there is a trend of disease-specific POMs to assess the various morbidities associated with individual conditions. To illustrate, MDASI is a POM tailored to AML/ MDS, specifically evaluating malaise, diarrhoea, muscle weakness and skin symptoms associated to the condition [32]. While it is beneficial to evaluate symptoms unique to the patient cohort, these trends have inevitably reduced the adaptability of the POMs.

### Lack of quality indicators in structure and process of care

There are generally fewer QIs evaluating the quality of structure and process of care. This trend was observed in the review conducted by Aslakson *et al* [16], yet the disparity persists to date. Despite being the second most commonly assessed domain, the process of care was evaluated mostly by QIs within the category of rate of utilisation of PC services, instead of caregiver burden and EoL care screening. Of the 10 studies focusing on the structure of care, eight zeroed in on human resources, while only two assessed the organisational structure and facilities and equipment. It is paramount to address the research underactivity in these domains as their QIs are fundamental for stakeholders in the set-up and provision of palliative care services, especially in resource-limited LMICs. QIs focussing on the structure of care should quantify human resource, infrastructure and equipment available currently and in preparation of the future in order to plan workforce, allocate budget and organise the service in an optimal way. To complement it, QIs targeting at process of care should evaluate the utilisation of palliative care services across acute and community settings in the real world. Future work on health economics and implementation science using QIs in palliative care will be crucial in bridging the gap in the structure and process of care domains.

### Variability of evidence

Despite 70% of included studies having high-quality evidence according to the MMAT, certain research methodologies are underutilised in palliative care research. The criticisms of PC studies include the fact that they were predominantly descriptive with a wide variation in sample size and in outcome measures [63]. The various complex physical, psychological, social and spiritual problems faced by patients, families and service providers often made the design and conduct of palliative care research challenging. Issues of participant burden, sample heterogeneity, data attrition and ethics were well described [64].

In our review, only two RCTs were included and only one of which was graded as high quality and neither of which used a blinded outcome assessment [54, 60]. Of the non-randomised studies, most did not account for confounders and several had incomplete outcome data. On a side note, only five of the included studies were qualitative in nature. As the complexity of patients and caregivers’ experiences are often not accurately reflected in quantitative assessments and measures, qualitative studies are vital in providing insights into the subsequent development and validation of quantitative surveys.

### Recommendations

Given the lack of standardisation of QIs and POMs, the shortage of QIs in structure and process of care, as well as the variability of evidence, we recommend the development of a palliative care QI and POM repository, ideally set up by an accredited institution, such as the WHO, ESMO or NCP, to guide and frame research in palliative care. The existence of validated metrics will help align clinical practice, facilitate audit and accelerate research for clinicians, service provider and academics. Previous successful initiatives in Flanders and the Netherlands have focused on creating standardised POM sets, which apply to all palliative care settings and patient groups to allow for ease of comparison [12] While this is one approach, another could be to focus on specific disease populations, separating cancer from non-cancer NCDs to allow for easier modifications of standardised tools to assess disease-specific morbidities. MDASI which contains both a general instrument to assess quality of life in cancer patients and a dedicated section for AML cancer patients, is a good example [32]. In addition, a QI and POM repository would also facilitate the expansion of research into LMICs. Less than 10% of the studies (*n* = 3) in our review were from LMICs, highlighting a yawning geographical gap in palliative care research activity. This may be because most published QIs and POMs were developed in high-income settings and therefore did not accurately reflect the resources and treatment options available in many health systems in LMIC. Finally, as treatment capacity expands, the assessment tools used in LMICs will need to be equally dynamic [6]. A repository would be particularly useful in developing and disseminating tools for these settings.

In addition, we encourage researchers to explore alternative research designs that can be more feasible in real-world palliative care without compromising on its integrity and robustness. One example is the use of desirable alternative or delayed interventions for control groups, such as randomised fast-track or wait-list designs paired with intention-to-treat analysis. This design helps maintain group equivalency over time without compromising on ethics, as either the fast-track or wait-list arm ensures that all participants receive the treatment [64]. Other strategies include cluster sampling, narrative research and action research.

## Conclusion

The rise in global cancer incidence has led to a concomitant increase in demand for palliative care. However, despite an upturn in research, there is a lack of standardisation of quality indicators and patient outcome measures overall with limited evidence in various domains of palliative care. Henceforth, we recommend stakeholders co-develop a quality indicator and outcome measure repository to promote uniformity and equal representation of all aspects of palliative care. Emphasis should be on developing and validating QIs for the structure and process of palliative care which are currently understudied. We believe standardised and validated QIs and POMs will provide the foundation to build, monitor and evaluate palliative care services that are holistic for cancer patients and feasible for integration into health systems across all income settings.

## Conflicts of interest

The authors have no conflicts of interest to declare.

## Funding

RS and PN are funded by Medical Research Council Global Alliance of Chronic Disease Grant ACCI No GACD-025. RS and PN are also funded by BASO/ Rosetrees Research Grant in Cancer Surgery. The funders have no role in study design; in the collection, analysis, and interpretation of data; in the writing of the manuscript; and in the decision to submit the manuscript for publication.

## Figures and Tables

**Table 1.** Quality appraisal of all included studies using the 2018 mixed method appraisal tools.

Author, Year	Type of research					Overall score	Quality
**Qualitative**							
	Approach relevant to objectives	Data collection process adequate for objectives	Findings adequately derived from data	Analysis substantiated by data	Coherence		
Namisango *et al* [35]	Y	Y	Y	Y	Y	100%	High
Schurr *et al* [37]	Y	Y	Y	N (first phase, awaiting quantitative analysis)	Y	80%	High
Rosenblum et al* [21]	Y	N (small sample size, responder bias and social desirability bias)	Y	Y	Y	80%	High
Matthys *et al* [47]	Y	N (low response rate)	Y	Y	Y	80%	High
Cocks *et al* [41]	Y	Y	Y	Y	Y	100%	High
Quantitative randomised controlled trials							
	Sampling strategy relevant to objectives	Groups comparable at baseline	Complete outcome data	Blinded outcome assessment	Adherence to intervention		
Solar *et al* 54]	Y	Y	N (high dropout rate)	N	Y	60%	Medium
Marcolin *et al* [60]	Y	Y	Y	N	Y	80%	High
Quantitative non-randomised studies							
	Sample representative of population	Appropriate measure	Complete outcome data	Account for confounders	Intervention administered as intended		
Haroen *et al* [33]	Y	Y	Y	N	Y	80%	High
Yennurajalingam *et al* [32]	N (small sample size with high exclusion rate due to non-participation and time constraint)	Y	N (21.4% dropout rate)	N	Y	50%	Medium
Sun *et al* [36]	Y	Y	U (unable to clarify completeness of data)	Y	Y	80%	High
Rosenblum et al* [25]	N (a single academic centre and excluded outpatient)	Y	Y	N	Y	60%	Medium
Lee et al* [22]	N (high exclusion rate due to death, delirium, cognitive impairment)	Y	Y	N	Y	60%	Medium
Paschos *et al* [38]	Y	Y	Y	Y	Y	100%	High
Abuhammad et al [39]	Y	Y	Y	N	Y	80%	High
Mercadante *et al* [40]	Y	Y	Y	N	Y	80%	High
Boyd et al* [23]	Y	Y	Y	N	Y	80%	High
Schad *et al* [42]	Y	Y	Y	Y	Y	100%	High
Gensheimer et al* [24]	N (specific electronic patient record system not transferrable to other centres)	Y	Y	N	Y	60%	Medium
Hsiao *et al* [45]	Y	Y	Y	Y	Y	100%	High
Mercadante *et al* [49]	N (small sample size)	Y	Y	N	Y	60%	Medium
Soper *et al* [50]	N (single centre)	Y	Y	N	Y	80%	High
Bavelaar *et al* [51]	Y	Y	N (missing data)	Y	Y	80%	High
Jewitt et al* [27]	Y	Y	U (uncertain outcome due to different ways of billing)	Y	Y	80%	High
Robertson et al* [28]	N	Y	N (missing data)	Y	Y	60%	Medium
Pitman *et al* [53]	N (rural settings only)	Y	Y	N	Y	60%	Medium
Rodriguez-Gonzalez *et al* [56]	Y	Y	Y	N	Y	80%	High
Bos-van den Hoek *et al* [57]	N (small sample size)	Y	Y	N	Y	60%	Medium
Zapata *et al* [58]	Y	Y	Y	N	Y	80%	High
Pereira et al* [30]	Y	Y	Y	N	Y	80%	High
Cox *et al* [61]	Y	Y	Y	N	Y	80%	High
Islam *et al* [44]	N (small purposive sampling lacking generalisability)	Y	Y	N	Y	60%	Medium
Quantitative descriptive							
	Sampling strategy relevant to objectives	Sample representative of population	Appropriate measure	Low risk of non-response bias	Analysis appropriate for objectives		
Pedersen *et al* [34]	Y	Y	Y (validated measure)	N (34% non-response rate which could be contributed by symptoms)	Y	80%	High
Otani *et al* [43]	Y	Y	Y	Y	Y	100%	High
Li *et al* [46]	Y	Y	Y	Y	Y	100%	High
De Souza et al [48]	Y	Y	Y	Y	Y	100%	High
Ribeiro et al* [26]	Y	Y	Y	Y	Y	100%	High
McFerran et al* [29]	Y	Y	Y	Y	Y	100%	High
Farrell *et al* [59]	Y	N (lack community-based representation)	Y	N (non-standardised data collection)	Y	60%	Medium
Tabuyo-Martin et al* [31]	Y	Y	Y	Y	Y	100%	High
Xue *et al* [55]	Y	Y	U (lacking description of different attitudes)	Y	N (no data to substantiate cost related to palliative care)	60%	Medium
Mixed methods							
	Research design relevant to objectives	Integration of results relevant to objectives	Adequate interpretation of outputs	Adequate address of divergences and inconsistencies across results	Adherence to quality criteria of each tradition of methods involved		
Moore *et al* [52]	Y	Y	Y	Y	N (not a representative sample)	80%	High

The appraisal for each domain of the study is colour-coded- no (N): red; unknown (U): orange; and yes (Y): green

* represents studies related to quality indicators. If not labelled, the studies are related to patient outcome measures

## Appendix A

### Palliative care quality indicators search terms

Palliative care: ‘palliative care’ [MeSH Terms] OR (‘palliative’[All Fields] AND ‘care’[All Fields]) OR ‘palliative care’[All Fields] hospice care: ‘hospice care’[MeSH Terms] OR (‘hospice’[All Fields] AND ‘care’[All Fields]) OR ‘hospice care’[All Fields] end of life care: ‘terminal care’[MeSH Terms] OR (‘terminal’[All Fields] AND ‘care’[All Fields]) OR ‘terminal care’[All Fields] OR (‘end’[All Fields] AND ‘life’[All Fields] AND ‘care’[All Fields]) OR ‘end of life care’[All Fields] OR ‘hospice care’[MeSH Terms] OR (‘hospice’[All Fields] AND ‘care’[All Fields]) OR ‘hospice care’[All Fields] OR (‘end’[All Fields] AND ‘life’[All Fields] AND ‘care’[All Fields])

Cancer: ‘cancer’s’[All Fields] OR ‘cancerated’[All Fields] OR ‘canceration’[All Fields] OR ‘cancerization’[All Fields] OR ‘cancerized’[All Fields] OR ‘cancerous’[All Fields] OR ‘neoplasms’[MeSH Terms] OR ‘neoplasms’[All Fields] OR ‘cancer’[All Fields] OR ‘cancers’[All Fields] patients: ‘patient’s’[All Fields] OR ‘patients’[MeSH Terms] OR ‘patients’[All Fields] OR ‘patient’[All Fields] OR ‘patients’s’[All Fields]

Quality: ‘qualities’[All Fields] OR ‘quality’[All Fields] OR ‘quality’s’[All Fields] measures: ‘measurability’[All Fields] OR ‘measurable’[All Fields] OR ‘measurably’[All Fields] OR ‘measure’s’[All Fields] OR ‘measureable’[All Fields] OR ‘measured’[All Fields] OR ‘measurement’[All Fields] OR ‘measurement’s’[All Fields] OR ‘measurements’[All Fields] OR ‘measurer’[All Fields] OR ‘measurers’[All Fields] OR ‘measuring’[All Fields] OR ‘measurings’[All Fields] OR ‘measurment’[All Fields] OR ‘measurments’[All Fields] OR ‘weights and measures’[MeSH Terms] OR (‘weights’[All Fields] AND ‘measures’[All Fields]) OR ‘weights and measures’[All Fields] OR ‘measure’[All Fields] OR ‘measures’[All Fields] metrics: ‘benchmarking’[MeSH Terms] OR ‘benchmarking’[All Fields] OR ‘metrics’[All Fields] OR ‘metric’s’[All Fields] OR ‘metronidazole’[MeSH Terms] OR ‘metronidazole’[All Fields] OR ‘metric’[All Fields] patient satisfaction: ‘patient satisfaction’[MeSH Terms] OR (‘patient’[All Fields] AND ‘satisfaction’[All Fields]) OR ‘patient satisfaction’[All Fields] indicators: ‘indicate’[All Fields] OR ‘indicated’[All Fields] OR ‘indicates’[All Fields] OR ‘indicating’[All Fields] OR ‘indicative’[All Fields] OR ‘indicatives’[All Fields] OR ‘indicators and reagents’[Pharmacological Action] OR ‘indicators and reagents’[MeSH Terms] OR (‘indicators’[All Fields] AND ‘reagents’[All Fields]) OR ‘indicators and reagents’[All Fields] OR ‘indicator’[All Fields] OR ‘indicators’[All Fields] OR ‘indice’[All Fields] OR ‘indices’[All Fields]

28 Oct 2023

## Appendix B

**Table S1.** Summary table of all included palliative care quality indicator studies.

Country	Author	Study title	Funding	Methodology	Research design	Sample size	Pathology	Aim of study	Outcome of study	Type of palliative care service	Domain of palliative care	Quality indicators
USA	Rosenblum *et al* [21]	Institution of standardised consultation criteria to increase early palliative care utilisation in older patients with acute leukemia	N/A	Quantitative	Non-randomised, quality improvement (PDSA)	106	Cancer (Haematological)	To increase early PC utilisation by older patients with newly diagnosed AL using a standardised criteria to trigger inpatient PC consultation	The baseline PC consultation rate before our intervention was 55%. This increased to 77% and 80% during PDSA cycles 1 and 2, respectively. The median time from diagnosis to first PC consult decreased from 49 days to 7 days. Among patients who received a triggered PC consult, 43% had no subsequent inpatient or outpatient PC encounter after discharge.	Inpatient Care	Process	Frequency of PC consult
South Korea	Lee *et al* [22]	Outcomes of an acute palliative care unit at a comprehensive cancer centre in Korea	N/A	Quantitative	Non-randomised, observational	205	Cancer	To evaluate the first-year outcomes of the patients admitted to an acute palliative care unit (APCU) at a tertiary hospital in Korea	Among those who completed the ESAS, there were significant improvements in scores in the following symptoms: fatigue, depression, loss of appetite and shortness of breath. Physical symptoms (pain, fatigue, nausea, drowsiness, appetite and shortness of breath) and the total ESAS scores were significantly improved (*p* = 0.002 and *p* = 0.005, respectively). Each non-medical palliative care program, such as art and music therapy, yoga, foot massage, haircut and body care, showed no significant differences between the group who received them and those who did not.	Inpatient Care	Process	APCU Length of stay, APCU discharge rate
USA	Boyd *et al* [23]	Implementing a standardised workflow process to increase the palliative care to hospice admission rate		Quantitative	Non-randomised, quality improvement	N/A	General	To determine whether standardising the workflow process with chart completion leads to increases in the hospice admission rate for palliative care patients transitioning to hospice care	The palliative care to hospice admission rate increased by 11.5% in the postintervention group. The Electronic Medical Record (EMR) chart deactivation rate increased by 55.3%, which was statistically significant (*p* ≤ 0.001).	Hospice	Process	Hospice admission rate
USA	Gensheimer *et al* [24]	Use of machine learning and lay care coaches to increase advance care planning conversations for patients with metastatic cancer	N/A	Quantitative	Non-randomised, quality improvement	1,251	Cancer	To improve advanced care plan (ACP) using a computer model to select high-risk patients, with shorter predicted survival, for conversations with providers and lay care coaches.	35% of intervention clinic patients had ACP documentation compared with 3% of control clinic patients. Providers' prognosis documentation rate also increased in intervention clinics after the intervention (2%–27% in intervention clinics, *p* < 0.0001; 0%–1% in control clinics).	Outpatient Care (Clinic)	Outcome (Ethics and Legal)	Advanced care plan documentation rate, prognosis documentation rate
USA	Rosenblum *et al* [25]	National survey using CFIR to assess early outpatient specialty palliative care implementation	National Institutes of Health and the McElhattan Foundation	Qualitative	Survey	40	Cancer	To identify the facilitators and barriers to early Outpatient Specialist Palliaitve Care (OSPC) implementation and associated clinic characteristics	The most commonly agreed upon barriers to early OSPC included inadequate number of OSPC providers (73%), lack of performance metric goals (65%), insufficient space to deliver early OSPC (58%), logistical challenges created by early OSPC (55%) and absence of formal interdisciplinary communication systems (53%).	Outpatient Care (Clinic)	Structure (Human resource, Organisational), process	Time from advanced cancer diagnosis to palliative care input, number of palliative care providers, presence of multidisciplinary communication system
USA	Ribeiro *et al* [26]	Opioids and constipation therapy in the last week of life: their impact on patients, caregivers and the location of death	N/A	Quantitative	Descriptive, observational	127	General	To evaluate the impact of constipation on symptomatic control and patients' overall quality of life at this stage; to investigate whether constipation and caregiver fatigue is related to the place of death (hospital versus home)	82.6% of patients wished to die at home (occured in 74% of cases); constipation prevention protocol reduced constipation by 55.1%; morphine is more related with constipation and tapentadol seems to reduce constipation induced by opioids. Patients tended to die in hospitals when their caregivers were exhausted. Constipation in the last week of life does not seem to influence the well-being of patients or their caregivers significantly and the individualisation of intensive treatment of constipation is needed. Different opioids have different probabilities of causing adverse effects such as constipation. Future special support mechanisms can be created and activated for the most tired caregivers to avoid exhaustion and promote death at home, if that is the patient's will.	Home care	Outcome (Social), Process	Percentage of patients dying in a place of preference, Zarit Scale (caregiver burden)
Canada	Jewitt *et al* [27]	The effect of specialised palliative care on end-of-life care intensity in AYAs with cancer	Stronach Regional Cancer Centre, Newmarket, Ontario, Pediatric Oncology Group of Ontario, ICES (Institute for Clinical Evaluative Sciences)	Quantitative	Non-randomised, observational	7,122	Cancer	To evaluate whether specialised palliative care (SPC) had an impact on the intensity of end of life (EOL) care received by adolescents and young adults (AYAs) with cancer; to determine which subpopulations are at highest risk for reduced access to SPC	30% of AYAs received SPC and 13% received general palliative care (GPC). AYAs who died in earlier years, those with haematologic malignancies, males and rural AYAs were least likely to receive SPC. No PC involvement was associated with higher odds of receiving HI-EOL care. SPC involvement was associated with the lowest risk of HI-EOL care and decreased odds of ICU admission. SPC involvement was associated with the lowest risk of HI-EOL (high intensity end of life) care in AYAs with cancer. However, access to SPC remains a challenge.	Inpatient/ Acute care	Process	Rate of specialised palliative care consultation, intravenous chemotherapy <14 days from death, number of ED visit hospitalisation or any ICU admission <30 days from death
Canada	Robertson *et al* [28]	Association between consultation by a comprehensive integrated palliative care program and quality end-of-life care in patients with advanced cancer in Edmonton, Canada	Dorothy Jean Usher Memorial Summer Research Award	Quantitative	Non-randomised, observational	1,414	Cancer	To assess the impact of palliative care (PC) consultation on aggressive care at the end of life (EOL) within a comprehensive integrated PC program.	78.6% of eligible patients received PC consultation. PC consultation was independently associated with lower odds of ≥1 aggressive EOL care indicator. PC consultation >3 versus ≤3 months before death had a greater effect on lower aggressive EOL care. PC consultation is associated with less aggressive care at the EOL for patients with advanced cancer.	General	Process	Emergency room visits, hospitalisation, ICU admission and chemotherapy administration in the last 30 days of life and hospital death
Northern Ireland	McFerran *et al* [29]	Cost consequences of unscheduled emergency admissions in cancer patients in the last year of life	Public Health Agency of Northern Ireland, Cancer Focus Northern Ireland and Health Data Research UK, Macmillan-NI Cancer Registry Partnership (2016)	Quantitative	Descriptive, observational	3,134	Cancer	To examine the utilisation of unscheduled emergency end-of-life healthcare and estimates expenditure in this domain. To explore care patterns and quantify the likely benefits from service reconfigurations which may influence rates of hospital admission and deaths.	The highest service use and total cost was in those diagnosed at stage IV (38.4%), who required 22,099 days of care, costing £9,629,014. Palliative care support, identified in 25.5% of patients, contributed £1,322,328. A 3-day reduction in the mean length of stay with a 10% reduction in admissions, could reduce costs by £7.37 million. Regression analyses explained 41% of length-of-stay variability.	Inpatient/ Acute Care	Structure (Facilities and Equipment)	Cost of unscheduled emergency end-of-life healthcare, number of acute hospital bed days
Singapore	Pereira *et al* [30]	Integrated palliative homecare in advanced dementia: reduced healthcare utilisation and costs	Temasek Foundation Cares CLG Limited, Ministry of Health of Singapore	Quantitative	Non-randomised, Observational	323	Dementia	To determine the economic benefit of an integrated home-based palliative care programme for advanced dementia (Programme Dignity), evaluation is required. This study aimed to estimate Programme Dignity's average monthly cost from a provider's perspective; and compare healthcare utilisation and costs of programme patients with controls, accounting for enrolment duration.	Programme Dignity for advanced dementia reduces healthcare utilisation and costs. If scalable, it may benefit more patients wishing to remain at home at the end-of-life, allowing for a potentially sustainable care model to cope with rapid population ageing. It contributes to the evidence base of advanced dementia palliative care and informs healthcare policy making. Future studies should estimate informal caregiving costs for comprehensive economic evaluation.	Home Care	Structure (Facilities and Equipment)	Cost of home-care palliative care service
USA	Tabuyo-Martin *et al* [31]	Palliative medicine referral and end-of-life interventions among racial and ethnic minority patients with advanced or recurrent gynecologic cancer	N/A	Quantitative	Descriptive, observational	186	Cancer (Gynaecological)	To assess differences in palliative medicine referrals and end of life interventions (within the last 30 days of life) by race and ethnicity in a diverse population of gynecologic oncology patients.	Race was associated with variation in interventions and healthcare utilisation near end-of-life. Understanding the etiologies of these differences is crucial to inform interventions for care optimisation as it relates specifically to the health of minority patients.	Inpatient/ Acute care	Process	Palliative care referral rate, chemotherapy in the last 30 days of life, frequency of ED visit

**Table S2.** Summary table of all included palliative care patient outcome measure studies.

Country	Author	Study title	Funding	Methodology	Research design	Sample size	Pathology	Aim of study	Outcome of Study	Type of palliative care service	Domain of palliative care	Quality indicators
USA	Yennurajalingam *et al* [32]	Treatment of cancer-related-fatigue in acute hematological malignancies: results of a feasibility study of behavioural	Pfize, AbbVie, Genentech, Eli Lilly, Cellectis, Calithera, Ablynx, Stemline Therapeutics, Agios, Ascentage, Astra Zeneca, 47, Reata Pharmaceutical.	Quantitative	Non-randomised, observational	27	Cancer (Haematological)	To determine the feasibility of cognitive behavioural therapy (CBT) for cancer related fatigue in haematogical malignancies	The use of CBT was feasible with improvement of cancer-related fatigue, sleep quality and anxiety scores in HM.	Outpatient Care (Clinic)	Outcome (Physical, psychological, spiritual)	Change in functional assessment of cancer illness therapy (FACIT) - Fatigue, FACT-G, pittsburgh sleep quality index (PSQI), hospital anxiety depression scale (HADS), M.D. Anderson symptom inventory – acute myeloid leukemia (MDASI-AML/MDS), Herth Hope Index (HHI)
Indonesia	Haroen *et al* [33]	Knowledge and attitude toward end-of-life care of nursing students after completing the multi-methods teaching and learning palliative care nursing course	Directorate Research and Community Engagement of Universitas Padjadjaran, Indonesia	Quantitative	Non-randomised, observational	165	General	To assess the palliative care nursing (PCN) knowledge and attitude of nursing students toward end-of-life care (EoLC) after completing the multi-methods PCN courses.	PCN courses with multi-method learning and teaching are effective to increase PCN knowledge, particularly in pain and symptom management knowledge and also effective in increasing the positive attitudes toward EoLC.	General	Structure (Human resource)	Frommelt attitudes toward care of the dying (FATCOD)
Denmark	Pedersen *et al* [34]	Quality of life and mental health in real-world patients with resected stage III/IV melanoma receiving adjuvant immunotherapy	Herlev and Gentofte Research Counsil, The National Board of Health Denmark	Quantitative	Descriptive, observational	271	Cancer (Melanoma)	To investigate how HRQoL was affected during and after adjuvant immunotherapy in a real-world setting	Adjuvant nivolumab may affect some aspects of QoL, but the influence seems temporary. Patient characteristics, such as civil status, employment status and comorbidity were associated with HRQoL.	Outpatient Care (Clinic)	Outcome (Physical, psychological, spiritual)	European Organisation for the Research and Treatment of Cancer Quality of Life Questionnaire core 30 version 3 (EORTC QLQ-C30), Multidimensional fatigue inventory (MFI), Major depression inventory (MDI), concerns about cancer recurrence questionnaire (CARQ-4), decision regret scale (DRS), CAT cognitive functioning
Uganda	Namisango *et al* [35]	The meaning of healing to adult patients with advanced cancer	National Institutes of Health Clinical Centre, Uganda	Qualitative	Phenomenology, Interview	35	Cancer	To explore the meaning of the term healing from the perspective of adult patients with advanced cancer.	Themes from patients’ responses suggest subjective and varied definitions of healing which encompass physical, social, spiritual and psychological domains of wellbeing, distinct from the physical cure of disease.	Hospice	Outcome (Psychological, social, spiritual)	NIH-HEALS
Japan	Sun *et al* [36]	Evaluation of enhanced home care support clinics regarding emergency home visits, hospitalisation and end-of-life care: a retrospective cohort study in a city of Japan	Ministry of Health, Labor and Welfare, Japan	Quantitative	Non-randomised, observational	802	General	To evaluate whether enhanced HCSCs fulfilled the expected role in home healthcare	Enhanced HCSCs are more likely to be able to handle emergency home visits and end-of-life care at home.	Home Care	Process	Emergency home visits
Austria	Schurr *et al* [37]	Patient-reported outcome measures for physical function in cancer patients: content comparison of the EORTC CAT Core, EORTC QLQ-C30, SF-36, FACT-G and PROMIS measures using the International Classification of Functioning, Disability and Health	EORTC Quality of Life Group	Qualitative	Descriptive	-	Cancer	To compare content of frequently used PRO measures for PF in cancer patients	The results provide information about conceptual differences between common PRO measures for the assessment of PF in cancer patients. Our results complement quantitative information on psychometric characteristics of these measures and provide a better understanding of the possibilities of establishing common metrics.	General	Outcome (Physical)	EORTC CAT, EORTC QLQ-C30, SF-36, PROMIS physical function, FACT-G physical well-being scale
Netherlands	Paschos *et al* [38]	Are gastrointestinal problems, nutritional care and nutritional care needs associated with quality of life in patients with advanced cancer? Results of the observational eQuiPe study	Roparun Foundation	Quantitative	Non-randomised, observational	540	Cancer	To assess the association of gastrointestinal problems, received nutritional care and nutritional care needs with quality of life (QoL) in patients with advanced cancer.	Many patients with advanced cancer experience gastrointestinal problems, while only few patients receive nutritional care. These gastrointestinal problems, nutritional care needs and nutritional care are associated with lower QoL, probably due to reversed causality or the irreversible nature of these problems in the palliative phase.	General	Outcome (Physical)	EORTC QLQ-C30
Jordan	Abuhammad *et al* [39]	The efficacy of educational interventions on neonatal intensive care unit nurses’ knowledge and attitude toward neonatal palliative care	N/A	Quantitative	Non-randomised, observational	164	General	To examine the efficacy of an educational program in improving nurses’ knowledge and attitude toward Neonatal Palliative Care (NPC)	The NPC educational program is beneficial in improving nurse knowledge and attitudes for NPC services, as well as providing an effective educational program for nurses.	General	Structure (Human resource)	NiPCAS (evaluation of neonatal nurses’ attitudes)
Italy	Mercadante *et al* [40]	Maddalena opioid switching score in patients with cancer pain	N/A	Quantitative	Non-randomised, observational	106	Cancer	To assess an integrated score (Maddalena Opioid Switching Score) as a simple and repeatable tool to evaluate the outcomes of OS, facilitating the interpretation and comparison of studies and information exchange among researchers.	The Maddalena Opioid Switching Score significantly decreased after OS and was highly correlated to PGI of improvement (*p* < 0.0005). In patients with unsuccessful OS, no significant changes in the Maddalena Opioid switching score and patient global impression (PGI) were observed. A significant reduction in Edmonton symptom assessment scale items intensity was observed after OS. The Maddalena opioid switching score resulted to be a sensitive instrument for measuring the clinical improvement produced by OS.	General	Outcome (Physical, psychological)	Edmonton symptom assessment scale (ESAS)
UK	Cocks *et al* [41]	Content validity of the EORTC quality of life questionnaire QLQ-C30 for use in cancer	EORTC Quality of Life Group	Qualitative	Interview	113	Cancer	To evaluate the content validity of the QLQ-C30 for use with cancer patients.	The QLQ-C30 demonstrates good evidence of content validity for the assessment of functional health, symptom burden and health-related quality of life in patients with localised-to-advanced cancer.	General	Outcome (Physical, psychological, social)	EORTC QLQ-C30
Germany	Schad *et al* [42]	Evaluation of quality of life in lung cancer patients receiving radiation and Viscum album L.: a real-world data study	Iscador AG Arlesheim, Switzerland; Abnoba GmbH Pforzheim, Germany; Helixor Heilmittel GmbH Rosenfels, Germany	Quantitative	Non-randomised, observational	112	Cancer (Lung)	To analyse the changes in QoL of LC patients being treated with radiation according to oncological guidelines and add-on VA treatment in a real-world setting.	Add-on VA therapy reveals supportive effects for the QoL of LC patients. Particularly in combination with radiation a significant reduction in pain and nausea/ vomiting has been observed.	Home care	Outcome (Physical, social)	EORTC QLQ-C30
Japan	Otani *et al* [43]	Impact of taste/smell disturbances on dietary intakes and cachexia-related quality of life in patients with advanced cancer	SASAKAWA Health Foundation and JSPS KAKENHI	Quantitative	Descriptive, observational	378	Cancer	To investigate the impact of taste and smell disturbances on dietary intakes and cachexia-related quality of life (QOL) in patients with advanced cancer.	More severe taste and smell disturbances were associated with poorer dietary intakes and cachexia-related QOL. Diagnosing and treating such disturbances may improve dietary intakes and cachexia-related QOL, regardless of performance status and cachexia.	General	Outcome (Physical)	Functional assessment of Anorexia/Cachexia therapy Anorexia Cachexia subscale (FAACT ACS)
Bangladesh	Islam *et al* [44]	Coping strategy among the women with metastatic breast cancer attending a palliative care unit of a tertiary care hospital of Bangladesh	Bangabandu Skeikh Mujib Medical University, Dhaka, Bangladesh	Quantitative	Non-randomised, observational	95	Cancer (Breast)	To explore the different coping strategies adopted by the women with metastatic (stage IV) breast cancer attending the palliative care unit and their relationship with the common mental health issues	Different coping strategies, especially positive coping helps the patients to adapt with their disease over time. All women suffering from breast cancer should be routinely screened and assessed for psychological distress and ensure early intervention and management to promote a better quality of life.	General	Outcome (Psychological)	Hospital depression and anxiety scale (HADS), Brief COPE inventory
Taiwan	Hsiao *et al* [45]	Development of a scale of Nurses' competency in anticipatory grief counseling for caregivers of patients with terminal cancer	Chang Gung Medical Foundation	Quantitative	Non-randomised, observational	252	Cancer	To assess and manage the caregivers’ psychological problems, which in turn affects the caregivers’ quality of life, using the anticipatory grief counseling competency scale	The AGCCS can be used to evaluate the competency for improving caregivers’ quality of care. It can also facilitate in-service education planning and evaluation.	Caregiver support	Structure (Human resource)	Anticipatory grief counseling competency scale (AGCCS)
China	Li *et al* [46]	Development and assessment of a nutrition literacy scale for patients with end-stage kidney disease undergoing dialysis and its correlation with quality of life		Quantitative	Non-randomised, observational	208	End staged kidney disease	To construct a valid and reliable Nutritional Literacy Scale for patients with end-stage kidney disease (ESKD) receiving dialysis and evaluate associations between nutrition literacy and quality of life.	This new Nutrition Literacy Scale demonstrates high reliability and validity for Chinese ESKD patients undergoing dialysis. The nutrition literacy is influenced by age, education level, residence, occupational status and dialysis modalities, associated not only with nutritional status but also with quality of life.	General	Outcome (Physical)	Nutrition literacy scale (NLS)
Belgium	Matthys *et al* [47]	Is pre-bereavement collaboration between family caregivers and healthcare professionals associated with post-bereavement emotional well-being? A population-based survey	Flanders Innovation & Entrepreneurship	Qualitative	Survey	3,000	General	To investigate pre-bereavement collaboration with healthcare professionals and its association with emotional well-being of family caregivers of people with serious illness post-bereavement.	There is a positive association between perceived quality of collaboration at the end of life between healthcare professionals and family caregivers and post-bereavement emotional well-being of family caregivers; findings suggest the pertinence of attention from healthcare professionals to effective collaboration with family caregivers.	Bereavement support	Outcome (Psychological)	Positive and negative affect schedule (PANAS)
Brazil	De Souza *et al* [48]	Factors associated with the occurrence of adverse effects resulting from hypodermoclysis in older adults in palliative care: a cohort study	N/A	Quantitative	Descriptive, observational	127	General	To analyse the factors associated with local adverse effects resulting from hypodermoclysis in older adult patients in palliative care.	There was an incidence of 24% of adverse events, with catheter obstruction (11.3%) and swelling in the surrounding area of the hypodermoclysis site (8.5%) being the most frequent. Ondansetron administration by hypodermoclysis site was 3× more likely to have an adverse effect compared to not using this drug. A protective factor was evident with administration of 0.9% sodium chloride which contributed to the reduction of complications. The occurrence of adverse effects from hypodermoclysis in the study population was low.	Inpatient/ Acute care	Outcome (Physical)	Complication rate of subdermal administration of palliative care medication
Italy	Mercadante *et al* [49]	Breakthrough pain in patients with multiple myeloma: a secondary analysis of IOPS MS study	N/A	Quantitative	Non-randomised, observational	54	Cancer (Myeloma)	To characterise breakthrough pain (BTcP) in patients with multiple myeloma (MM)	54 patients with MM were examined; BTcP was more predictable (*p* = 0.04) compared to other tumours, with physical activity being the predominant trigger (*p* < 0.001). Patients with MM have their own peculiarities. Given the peculiar involvement of the skeleton, BTcP was highly predictable and triggered by movement.	Inpatient/ Acute care	Outcome (Physical)	Number of BTcP episodes, intensity, onset, duration, predictability, interference with daily activities, patient reported satisfaction with pain relief
USA	Soper *et al* [50]	The impact of Embedding a palliative care advance practice provider on a neuroscience intensive care unit service	N/A	Quantitative	Non-randomised, quality Improvement	1	General	To evaluate the impact of a full-time palliative care nurse practitioner on the neuroscience ICU team	The number of consults, family meetings and follow-up visits increased. Multidisciplinary staff members had a better understanding of the role of palliative care and there were many benefits from the addition of a palliative care nurse practitioner.	Inpatient/Acute care	Structure (Human resource), process	Presence of an inhouse palliative care nurse practitioner, number of palliative care consults, number of family meetings
UK	Bavelaar *et al* [51]	The impact of the mySupport advance care planning intervention on family caregivers' perceptions of decision-making and care for nursing home residents with dementia: pretest-posttest study in six countries	EU Joint Programme -Neurodegenerative Disease Research (JPND), Canadian Institutes of Health Research, the Czech Republic, Ministry of Education, Youth and Sport, Netherlands Organisation for Health Research and Development, Ireland Health Research Board, Alzheimer’s Society UK	Quantitative	Non-randomised, observational	88	Dementia	To investigate whether upscaling the intervention adapted to local context and complemented by a question prompt list impacts family caregivers' uncertainty in decision-making and their satisfaction with care across six countries; to investigate whether mySupport affects residents' hospitalisations and documented advance decisions	Family caregivers reported less decision-making uncertainty and more positive perceptions of care after the intervention. The number of advance decisions to refuse treatment was significantly higher after the intervention (21 versus 16); the number of other advance decisions or hospitalisations was unchanged. The mySupport intervention may be impactful in countries beyond the original setting	Caregiver Support	Outcome (Spiritual, ethics & legal), process	Rate of advance decisions to refuse treatment, number of hospitalisations, Decisional conflict scale (DCS) and Family perceptions of care scale (FPCS)
UK	Moore *et al* [52]	Exploring how family carers of a person with dementia manage pre-death grief: a mixed methods study	Alzheimer's Society, UK, Marie Curie, National Institute for Health Research Biomedical Research Centre, Camden and Islington National Health Service Foundation Trust	Mixed methods	Interviews and non-randomised, observational	150	Dementia	To assess the experience of pre-death grief for family carers of a person with dementia and to identify strategies to help carers manage pre-death grief.	Most carers identified multiple strategies for processing grief. Carers could readily identify supports and services that they found helpful for managing pre-death grief, yet current services appear under-resourced to meet growing demand.	Caregiver Support	Outcome (psychological), Process	Marwit-Meuser caregiver grief inventory short form, brief coping orientation to problems experienced (Brief-COPE) questionnaire
Australia	Pitman *et al* [53]	Triggering palliative care referrals through the identification of poor prognosis in older patients presented to emergency departments in rural Australia	N/A	Quantitative	Non-randomised, observational	235	General	This study aimed to estimate the usefulness of the criteria for screening and triaging to appropriate aLternative care (CriSTAL) tool in determining older patients' risk of death within 3-months after initial hospital admission.	A CriSTAL cut-off score of more than 7 yielded a sensitivity of 80.7% and specificity of 70.81% for a 3-month risk of death. Palliative care services were only used by 31% of the deceased in their last trimester of life. Prognostic tools provide a viable means of identifying individuals with a poor prognosis. Identification can trigger an earlier referral to palliative care, which will benefit the patient's wellbeing and quality of life.	Inpatient/ Acute care	Process	Criteria for screening and triaging to appropriate aLternative care (CriSTAL) Prognostic tool
Germany	Solar *et al* [54]	Screening versus multidimensional assessment of symptoms and psychosocial distress in cancer patients from the time of incurability	Innovation Funds of the German Federal Joint Committee.	Quantitative	Randomised, observational	504	Cancer	To compare two different strategies for detecting physical symptoms and psychosocial burden of patients with newly diagnoses incurable cancer and their effects on the further course of the disease.	A comprehensive, multidimensional assessment did not significantly differ from brief screening in preserving several dimensions of quality of life. These findings may positively influence the implementation of structured low-threshold screening programs for supportive and palliative needs in DKG certified cancer centres.	General	Outcome (Physical, psychological, Spiritual, social), process	FACT-G (Functional assessment of cancer therapy), NCCN (National Comprehensive cancer network) distress thermometer, IPOS (Integrated palliative care outcome scale), SCNS-SF-34-G (Supportive care needs Survey – short form), number of hospital days, utilisation of palliative care, utilisation of emergency service
China	Xue *et al* [55]	Attitudes and knowledge of palliative care of Chinese undergraduate nursing students: a multicentre cross-sectional study	N/A	Quantitative	Descriptive, observational	582	General	To investigate attitudes and knowledge toward palliative care among undergraduate nursing students in China and to explore correlations and associated factors.	The findings highlight the need to offer palliative care courses in nursing education and practice settings in Chinese health care settings. Nurse educators need to integrate the concept of palliative care into the curriculum of nursing education programs. Healthcare administrators and nurse leaders should promote investment and training in the education of nurses in practice settings to deliver high-quality palliative care services.	General	Structure (Human resource)	Frommelt attitude toward care of the dying scale (FATCODS)
Spain	Rodriguez-Gonzalez *et al* [56]	Using the emotional functioning in clinical practice to detect psychological distress in patients with advanced thoracic and colorectal cancer	FSEOM (Spanish Society of Medical Oncology Foundation) grant for Projects of the Collaborative Groups, Astra Zeneca	Quantitative	Non-randomised, Observational	639	Cancer (Thoracic and Colorectal)	To probe the usefulness of the emotional function (EF) subscale of the European Organisation for Research and Treatment of Cancer Quality of Life Questionnaire C30 (EF-EORTC-QLQ-C30) to assess psychological distress in cancer patients.	This study reveals that the EF-EORTC-QLQ-C30 subscale is a simple and effective tool for detecting psychological distress in people with advanced cancer.	Outpatient Care (Clinic)	Outcome (Psychological)	Emotional function (EF) of EORTC-QLQ-C30, Brief symptom inventory (BSI)-18 scales
Netherland	Bos-van den Hoek *et al* [57]	Blended online learning for oncologists to improve skills in shared decision making about palliative chemotherapy: a pre-posttest evaluation	Netherlands Organisation of Health Research and Development	Quantitative	Non-randomised, Observational	17	Cancer	To improve shared decision making (SDM) with advanced cancer patients, communication skills training for oncologists is needed. The purpose was to examine the effects of a blended online learning (i.e. e-learning and online training session) for oncologists about SDM in palliative oncological care and to compare this blended format with a more extensive, fully in-person face-to-face training format.	Blended online SDM training for oncologists was effective. However, the effects were smaller compared to face-to-face training. The availability of different training formats provides opportunities for tailoring training to the wishes and needs of learners.	General	Structure (Human resource)	Observing patient involvement scale (OPTION12)
USA	Zapata *et al* [58]	Honoring what we say we do: developing real-world tools for routine family caregiver assessment and support in outpatient palliative care	N/A	Quantitative	Non-randomised, Observational	736	General	The aim of this study is to develop an approach to conducting assessments of routine needs and support of family caregivers in outpatient palliative care practice using a quality improvement framework.	A majority of family caregivers reported moderate or severe distress related to caregiving (score ≥4 on a 10-point scale). The most common sources of distress included emotional distress, worry caregiving was negatively impacting their own health and planning for the future. Most caregivers reported feeling moderately or very well supported, most commonly by family, friends and faith/spirituality. Caregivers rated the supportive tool kit an 8.4 on a 10-point usefulness scale and 92% would recommend it to others. We successfully developed and piloted practical clinical tools for routine family caregiver screening and support.	Outpatient Care (clinic)	Structure (Human resource)	Caregiver needs assessment
USA	Farrell *et al* [59]	Associations between symptoms with healthcare utilisation and death in advanced cancer patients	SCELC, Statewide California Electronic Library Consortium	Quantitative	Descriptive, observational	817	Cancer	To identify factors related to healthcare utilisation and death in AOP.	ESAS pain, anxiety and total score were related to more PC visits (B = 0.31, 95% CI [0.21, 0.40], *p* < 0.001; B = 0.24 [0.12, 0.36], *p* < 0.001; and B = 0.038 [0.02, 0.06], *p* = 0.001, respectively). Total FACT-G score and physical subscale were related to total PC visits (B=−0.021 [−0.037, −0.006], *p* = 0.008 and B=−0.181 [−0.246, −0.117], *p* < 0.001, respectively). Lower FACT-G social subscale scores were related to more ER visits (B=−0.03 [−0.53, −0.004], *p* = 0.024), while increased tiredness was associated with fewer AC visits (B=−0.039 [−0.073, −0.006], *p* = 0.023). Higher total ESAS scores were related to death within 30 days (OR = 0.87 [0.76, 0.98], *p* = 0.027). The ESAS and FACT-G assessments were linked to PC and AC visits and death. These assessments may be useful for identifying AOPs that would benefit from routine PC.	General	Outcome (Psychological. Physical, social), Process	Edmonton symptom assessment scale (ESAS), Functional assessment of cancer therapy–general (FACT-G) scale
France	Marcolin *et al* [60]	The effects of foot reflexology on symptoms of discomfort in palliative care: a feasibility study	N/A	Quantitative	Randomised, observational	14	General	To assess the feasibility of FR in a population of inpatients in a palliative care unit (PCU). Its secondary objective was to assess the impact of an FR session on some symptoms of discomfort (anxiety, pain, troubled sleep and psychological distress).	This study confirms the feasibility of an FR session for patients hospitalised in a PCU. It resulted in a slight improvement in sleep quality. For other discomfort symptoms such as anxiety, pain and distress, FR yielded a non-significant improvement. Significant results would have needed a larger cohort.	Inpatient/ Acute care	Outcome (Physical, psychological)	ESAS sleep quality score
USA	Cox *et al* [61]	Trajectories of palliative care needs in the ICU and long-term psychological distress symptoms	N/A	Quantitative	Non-randomised, observational	159	General	To describe trajectories of palliative care needs during ICU care and to determine if changes in needs over 1 week was associated with similar changes in psychological distress symptoms at 3 months.	Serious palliative care needs were common and persistent among families during ICU care. Improvement in needs was not associated with less psychological distress at 3 months. Serious needs may be commonly underrecognised in current practice.	Inpatient/Acute care	Process	Needs at the end-of-life screening tool (NEST)
